# Children require more evidence to revise beliefs under gradual perceptual change

**DOI:** 10.1007/s00426-026-02319-0

**Published:** 2026-06-03

**Authors:** Elisabeth Stöttinger, Beate Priewasser, Eva Rafetseder

**Affiliations:** 1 University of Sustainability - Charlotte Fresenius Privatuniversität, Christine-Touaillon- Straße 11/1, Vienna, 1220 Austria; 2 University Hospital of Paediatrics and Adolescent Medicine, Salzburg, 5020 Austria; 3 University Hospital of the Paracelsus Medical Private University, Salzburg, 5020 Austria; 4 University of Stirling, Stirling, FK9 4LA Scotland

**Keywords:** Belief revision, Local vs. global processing, Visual imagery

## Abstract

Rafetseder et al. (2021) were the first to demonstrate that children have difficulty revising their beliefs when objects morphed from one object (e.g. a rabbit) into another (e.g. a duck). Children aged four to eight reported the second object significantly later than adults. This is surprising, because both children and adults were presented with the same initial information (“it is a rabbit”) and were exposed to the same contradictory visual evidence. We conducted three experiments to replicate the results and to and explore reasons for children’s delayed belief revision. In Experiment 1 (*N* = 58; 3- to 6-year-olds), we demonstrated that children only struggle to identify the second object in the gradual condition, while displaying adult-like categorical perception when the same images were presented individually outside of the morphing context. In Experiment 2 (*N* = 86; 6- to 11-year-olds), we found that only children aged 9 years and over showed a similar pattern to adults, and that a global processing style facilitated identification of the second object. In Experiment 3 (*N* = 47, 5- to 6-year-olds), when children were explicitly informed of the two possible interpretations of the morphed images, their performance improved, although not to the level observed in the individual condition of Experiment 1. These findings suggest that children of different ages struggle to revise their beliefs in a gradually changing environment for different reasons. While older children may use less efficient, potentially local, exploration strategies, younger children may have difficulties with mental imagery.

**Supplementary Information:**

The online version contains supplementary material available at 10.1007/s00426-026-02319-0.

The ability to revise a belief is a critical human skill that facilitates adaptive behaviour in an ever-changing and unpredictable environment. While belief revision can be triggered by surprising and unexpected events (e.g., a sudden flash of lightning), it often results from the accumulation of gradual changes in a noisy and uncertain environment (e.g., the gradual accumulation of dark clouds). Despite a constantly changing environment, we tend to interpret the world categorically. For example, we might say, ‘The weather has changed’, ‘The steak is now cooked to perfection’ or ‘The mood has shifted’. While the reaction to change can be categorical, the stimuli they depend on vary continuously (e.g. the accumulation of clouds, the colour of the meat or slight shifts in the curve of a lip). As this type of belief revision is crucial for social interaction and even survival (Calder et al., 1996; Etcoff & Magee, 1992; Grinband et al., 2006; Hartendorp et al., 2010; McCullough & Emmorey, 2009; McGuire & Kable, 2015), it is important to understand how the ability to revise beliefs in response to subtle changes in visual stimuli develops.

Developmental research agrees that early on children reliably adjust their beliefs when presented with contradictory evidence even when they are confident in their own knowledge (Hagá & Olson, 2017a) and particularly when they receive support (Bonawitz et al., 2012). For example, children from age 4 adjust their beliefs according to their own direct perceptual access (i.e., what they see) and that of a third person (Köymen & Tomasello, 2018; Miosga et al., 2020), and depending on how counterintuitive information is presented to them (Lane et al., 2014). Early on children – like adults (Tenenbaum et al., 2006) – already revise their beliefs in line with Bayesian learning principles. According to the Bayesian framework we start with a set of beliefs about which structures are likely prior to observing any data (i.e. our ‘priors’). When confronted with new data, we evaluate the probability of this data given our priors (i.e., ‘likelihood’). Whether or not we revise our beliefs when the data contradicts our priors depends on (1) the strength of the initial belief, (2) the strength of the data, and (3) our ability to integrate the two (Bonawitz et al., 2012; Gopnik & Bonawitz, 2015 for a review). Following Bayesian principles children are more likely to revise their beliefs when the counterevidence is stronger than their initial belief, but less likely when the counterevidence is weaker (Kimura & Gopnik, 2019; Langenhoff et al., 2023). Similarly, children are more likely to revise their beliefs when their initial beliefs are weak (Lucas et al., 2014) and less likely when their initial beliefs are strong (Schulz et al., 2007). Children’s belief revision improves when they are trained to either question their initial belief or acknowledge the strength of contradictory evidence (Bonawitz et al., 2012). Together, these studies suggest that both young children and adults revise their beliefs in ways that are consistent with Bayesian principles.

Surprisingly, however, Rafetseder et al. (2021) found that children up to 9 years of age revised their belief in a gradually changing environment significantly later than adult participants although the dependence on priors was removed and the strength of evidence was systematically controlled. In their study participants were presented with a picture morphing paradigm where objects gradually morphed over 15 iterations from an unambiguous object (e.g., rabbit) to a different unambiguous object (e.g., duck). In this task, participants were shown an image (e.g. a rabbit) to identify the object, therefore eliminating the need for prior knowledge. The perceptual evidence was then gradually varied along a morph continuum between the two categories (e.g., 0% animal A → 100% animal B), thereby gradually increasing the likelihood of the second interpretation.

Rafetseder et al. (2021) found that children aged 4 to 8 years reported the second object significantly *later* than adults. Since both groups were exposed to the same initial picture (e.g. a rabbit) and the same gradual changes in the pictures, the ability to identify the second object in this picture morphing task may depend on emerging factors. One hypothesis is that children’s delayed ability to revise their beliefs in response to subtle changes in visual stimuli may be due to their difficulty in generating new hypotheses, which may arise for at least two reasons:

For efficient generation of potential alternative interpretations participants *first* need to visually process the morphed stimuli in a certain way. Rafetseder et al. (2021) argued that focusing on small local changes in these tasks does not support recognition of the new object. For example, if a rabbit is morphing into a cat, noticing the change of local details (e.g., the rabbit’s ears are getting smaller and the tail is getting longer), does not support recognition of the cat. Instead, only participants who adopt a more global (holistic) processing style (i.e., perceiving the overall structure or gestalt of an image before focusing on specific details) should be able to revise their beliefs efficiently. In fact, preliminary eye-tracking data from Rafetseder et al. (2021) showed that children who focused mainly on one detail of the picture (e.g., the animal’s eye) identified the second object later than children who allocated their attention to wider clusters of the picture. This is in line with studies showing that the transition from a local to an ‘adult-like’ global bias is happening relatively late in childhood, between 7 and 9 years (Martens et al., 2014; Nayar et al., 2015; Poirel et al., 2008) or even adolescence (Moses et al., 2002; Scherf et al., 2009; but see Vinter et al., 2010).

*Secondly*, successful exploration requires participants to actively search for an alternative interpretation (e.g., “It’s a rabbit at the moment, but what else could it be?“). For that participants must be able to recognise that not all interpretations are equally plausible, as the new interpretation must be compatible with the actual stimulus. This requires a participant to be able to generate templates of potential objects and project them onto the current item in order to recognise whether an object is a potential match. This cognitive ability is known as mental imagery – an ability for which the most substantial development occurs between the ages of 8 to 14 years (see Burnett Heyes et al., 2013 for a review; Kosslyn, 1996; Kosslyn et al., 2006).

As Rafetseder et al. (2021) were the first to show that children revise their beliefs significantly later than adults in response to subtle visual change, we conducted three experiments to replicate this effect and investigate potential reasons underlying it.

In Experiment 1, we focussed on replicating the effect. This issue is not trivial, particularly given the ongoing debate surrounding replication failures in psychology (Bohannon, 2015). As the finding by Rafetseder et al. directly contradicts previous research showing that children *are* able to adjust their beliefs when presented with contradictory evidence at an early age (Bonawitz et al., 2012; Gopnik & Bonawitz, 2015; Hagá & Olson, 2017a; Kimura & Gopnik, 2019; Köymen & Tomasello, 2018; Lane et al., 2014; Langenhoff et al., 2023; Miosga et al., 2020; Schulz et al., 2007), it is important to assess its robustness.

In Experiment 2, we addressed the authors’ suggestion that a local processing style could account for the variance observed in the picture morphing task. Based on the hypothesis that a more global processing style facilitates belief revision in the context of visual change, we predicted that children with a more global processing style should be able to recognise the second object in the picture morphing task earlier. We also expanded the age range in Experiment 2 to establish at what age children begin to exhibit a pattern of results similar to those of adults.

In Experiment 3, we eliminated the need for mental imagery by asking children to sort picture cards in boxes displaying the two possible interpretations (e.g., a rabbit or a cat). This allowed children to directly compare the card with each option rather than generating alternatives through mental imagery themselves. We hypothesised that if children struggle to revise their beliefs in the picture morphing task due to an inability to use mental imagery to form new hypotheses, eliminating the need for exploration should significantly improve their performance.

## Experimental 1

The aim of Experiment 1 was to replicate the results of Rafetseder et al. (2021), while taking care to control for potential confounds.

*First*, Rafetseder et al. (2021) used impoverished and somewhat ambiguous stimuli that allowed for interpretations other than the ones intended by the experimenters. For example, when a cat turned into a swan, children (and adults) named other animals (e.g., a dinosaur). It may be that the stimulus material was simply too ambiguous, which could have been especially disadvantageous for younger children, who are known to perseverate when faced with ambiguity (Doherty & Wimmer, 2005; Gopnik & Rosati, 2001; Rock et al., 1994; Wimmer et al., 2011). To rule out that the delay was the result of the ambiguous nature of the stimulus material we used a different selection of picture sets for which approximately 700 healthy adults rarely reported any other objects than the first or the second object (Stöttinger et al., 2016). With the selection of only categorically perceived sets, response options were limited to only two interpretations.

**Fig. 1:**
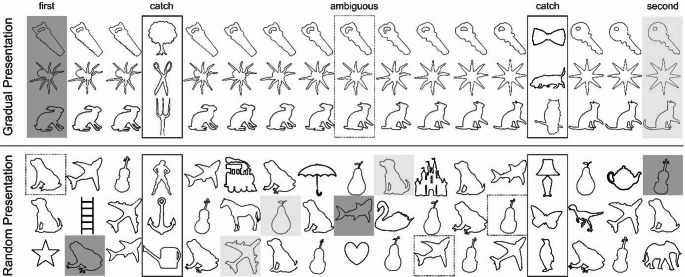
Picture morphing materials. Note: Three picture sets were presented in a gradually morphing context (top graph). Pictures morphed over 15 iterations from the first (dark grey) through an ambiguous object (dotted square) into a second object (light grey). In the random condition (bottom graph) pictures of three sets were shuffled and presented in a random order (e.g., eights picture of the frog-dog picture set followed by the ninths picture of the shark-plane picture set). For the analysis, ratings in the random condition were put back into the initial order (e.g., from 100% violin to 100% pear) and were then compared with the ratings for the same pictures presented in the gradual condition but from different participants. Catch trials were presented at the fourth and fourteenth position (solid square). Picture sets were excluded if a child perseverated on a single response in these catch trials. Fifteen additional pictures of unrelated objects were added in the random condition (e.g., train, umbrella, castle, etc.) but were not analysed further

Moreover, in addition to presenting half of the sets in a gradual condition, like Rafetseder et al. (2021) did, images of the other half of the sets were shuffled and presented in a random order (Fig. 1). The purpose of this random condition was to obtain an estimate of how children respond to each picture, independent of the evolving history of the series.

Although the pictures were presented in a random order rather than a stepwise order, the results with adult participants show that the pictures are still perceived in a categorical way (Stöttinger et al. 2016, 2018b). That is, despite the stepwise change in the actual ratio of the image, the image is typically interpreted according to its dominant interpretation. This results in an S-shaped curve for the probability of “reporting the second object” with the inflection point roughly in the middle (50% first object: 50% second object; Hartendorp et al., 2010; Stöttinger et al., 2016).

Interestingly, studies show that categorical perception emerges early in development. For example, Cheal and Rutherford (2011) randomly presented 3.5-year-olds with morphs of happy and sad facial expressions. Using an identification task (i.e., children had to categorise the face as either happy or sad) together with a discrimination task (i.e., two pictures were presented and the child had to decide whether the facial expression was the same or different), they showed that 3.5-year-olds categorized emotional facial expressions along the same boundaries as adults. Similarly, White (2016) demonstrated that even 3.5-month-old infants exhibit categorical perception, looking longer at image pairs crossing category boundaries (e.g., dog–cat morphs) than within-category pairs (e.g., two cats). These findings suggest that categorical perception, when limited to two options, is present from a very early age.

If children in Rafetseder et al. (2021) only had difficulties with the ambiguous nature of the stimulus material, we hypothesise that, given the improved stimulus material, they should now produce a comparable S-shaped curve of second object responses to an image in the gradual condition as they would in the random condition. However, if the delayed identification of the second object in the gradual condition indicates a genuine delay in belief revision, we expect that children will need more contradictory evidence to identify the second object in the gradual condition than in the random condition. In other words, we expect the S-curve to be shifted to the right, whereas the inflection point in the random condition should occur around the middle of the series.

*Second*, the morphing task requires individuals to understand and follow task instructions, thus demanding some level of verbal competence. It is possible that – despite noticing the changes between objects – children failed to follow the task instructions. The verbal instruction in the original task was abstract (“*One animal changes into another animal at a time*”), and younger children in particular may not have fully understood what they were supposed to do. In the current experiment, we therefore modified the instructions by using a cover story where “Freddy, the magic sheep” casts a spell on objects to change them. In two separate training series children were presented with the first object (e.g., bear), while the remaining fourteen pictures of the set were still hidden inside a paper cover. Children were told that Freddy would now transform the object into a completely different object. The paper cover was then slowly pulled away while children were repeatedly asked if they already knew what the bear was going to become (see Supplemental Materials for further details). Only children who named the new object eventually were included in the study.

*Third*, we evaluated whether the development of other domain-general cognitive abilities, such as Verbal IQ (WPPSI-III; Petermann, 2009), inhibition and cognitive flexibility (Kerns & McInerney, 2007), as well as remembering what the object was initially, could explain additional variance in task performance beyond that already accounted for by age. We hypothesised that, if the delay in reporting the second object was due to limited domain-general cognitive abilities, earlier reporting of the second object should correlate with a higher score in these measurements, even after controlling for age (see Supplemental Materials for a more detailed discussion of the rational to include these specific measures).

## Methods

### Participants

In this study we aimed for a similar sample size in each age group as tested in Rafetseder et al. (2021). A total of 58 children (37 girls) from three different age groups (3- and 4-year-olds: *n* = 20, *M*_age_= 50.05 ± 4.40 months; 5-year-olds: *n* = 20, *M*_age_= 65.40 ± 3.20; 6-year-olds: *n* = 18, *M*_age_=74.22 ± 1.96) participated in this study (*M*_age_= 62.84 ± 10.56 months). Six additional children were tested but excluded because they either quit the picture morphing task early (gradual: *n* = 1; random: *n* = 4) or did not show compliance (*n* = 1).

Children were recruited from three different nurseries. Each child was tested individually in two sessions (á 15 min) in a quiet room. Parents gave informed written consent to test and video-record their child and each child gave their assent prior to participation. Children received a diploma as well as a small gift for their participation regardless of whether they completed the experiment. This study followed the guidelines for developmental behavioural studies approved by the ethics committee of the University of Salzburg (“Outline application for the implementation of developmental psychology studies”; EK-GZ: 42/2016).

### Measures and procedure

Testing was done in two sessions with three tasks in each session: one picture morphing task (gradual or random condition), one control task (Verbal IQ or Go/No Go) and one False Belief task (one of two parallel versions). Data of the False Belief tasks was recorded for a different study and will be reported elsewhere. The order of the tasks was counterbalanced using a Latin Square Design, meaning that each task was eventually presented at one of the six positions.

### Picture morphing task

Six sets á 15 pictures were used (i.e., Frog-> Dog, Violin-> Pear, Shark-> Plane, Spider-> Sun, Rabbit-> Cat, Saw-> Key; https://osf.io/qi2vg/*).* Each picture contained the line drawing of a commonly known object or animal. Line drawings were printed on a white background (5.6 × 5.6 cm). All pictures were presented on a grey background at the centre of a 15.6inch screen. As soon as the child gave an answer the instructor pressed a button and a fixation cross appeared. A further button press released the next image.

Three sets were presented in a gradual order in which a known object morphed over fifteen iterations into a different object (Fig. 1). Three additional sets of pictures were presented in a random order. To mitigate the risk that participants might have perceived some pictures as being presented repeatedly, a set of fifteen distinctly different images (e.g., horse, elephant, etc.) was interspersed throughout the random presentation. These additional pictures were not analysed further. The order of the two conditions (gradual or random first), the assignment of sets (to gradual or to random) and the exact order of sets (order 1: Set1 - Set2 - Set3 or order 2: Set3 - Set2 - Set1) was varied between participants, resulting in eight different task sequences. For exploratory purposes, one of these gradual sets was presented again immediately after the gradual condition (i.e., repeat condition; see Supplemental Materials).

#### Catch trials

In all picture sets, two additional, non-related objects were presented at the 4th and 13th position (Fig. 1). These pictures served as catch trials to assess whether participants were simply perseverating. Only those picture sets were included in the analysis for which children named both catch trials correctly.

#### Vocabulary check

Following the procedure of Rafetseder et al. (2021), line drawings of the objects were presented on 9 × 8 cm paper cards *prior* to the actual task and children were asked to name each object. This was done to guarantee that all children were familiar with the names of both unambiguous objects of each set. If a child failed to identify an object, the name of the object was given and the card was presented again at the end of the vocabulary check.

#### Procedure

Each child was tested individually in a quiet room at the kindergarten. All test sessions were video-recorded. Answers in the picture morphing task were transcribed. Testing was carried out by two research assistants. To familiarise children with the experimenters, the research assistants went to the kindergarten to introduce themselves two weeks prior to testing. They used the hand puppet “Freddy, the magic sheep” to perform a magic trick. This was done to motivate children for participation and to introduce “Freddy” who played a critical role in the introduction of the gradual condition of the picture morphing tasks.

In the *Gradual Condition* children were asked if they remembered Freddy the magic sheep, who is enchanting pictures of objects and if they were curious about more tricks. Using two sets not presented in the actual test, children were then familiarised with the test procedure. During this training, the first slip included all fifteen images from the bear-pig set (https://osf.io/bkwxr), with only the bear visible at first. After asking the children if they recognised the bear, they were told that Freddy would magically transform it into something else. As the slip was slowly revealed, children were repeatedly asked if they could guess what the bear would become (“What is that going to be?”). Most children (75%) correctly identified the pig eventually. If they could not identify it, the answer was provided. Children were then asked if they wanted to see another trick. The second slip contained ten images, including nine from the fly-fir tree set (https://osf.io/vbfvt*)* and a “catch trial” image of a mushroom. One image at a time was revealed, with children again being asked to report what they saw (“What is it?”). After the answer of the child was confirmed by the instructor, the next picture was revealed and children were asked “Is it still a fly?”. After three fly pictures, the catch trial appeared, and the children were told that Freddy hid something else there and they should keep looking out for what the fly was going to be. Once a child identified the tree, the instructor said “It might be a tree. But let’s see if it really becomes a tree”. All children eventually identified the second object, showing they understood the task instructions. Finally, they were asked if they wanted to see more of Freddy’s transformations, but this time on the computer (“I’ll show you some more things that Freddy has enchanted. But this time on the computer. You should tell me for each picture what you see. Okay?”). Immediately after completion of a gradual set, children were asked whether they still remembered what the object was before Freddy had transformed it (*Explicit Memory question*). (See Supplemental Materials for more details together with the exact instruction).

In the *Random Condition* children were informed that they will see different pictures on the computer and that they should report what they saw.

#### Coding

Responses were coded as seeing the “first object”, seeing the “second object”, or seeing “a different object other than the first or second object”. Coding was done by two independent raters based on a list of validated picture terms from Stöttinger et al. (2016; https://osf.io/qi2vg/*)*, with an interrater agreement of 99.41%. Both raters were blind to the child’s age and performance in other tasks. The dependent variable was the picture position at which children reported the new object on average across three sets.

Only in very few individual cases (0.32% of all responses) children reported an object other than the first or second object, demonstrating that picture sets were perceived categorically by most children. In those cases, the position at which a child reported a different object was considered to be the position at which a child reported a second object. On rare occasions (0.50%) children’s responses indicated both objects at once (e.g., “shark turning into a plane”) with a similar frequency in the gradual (0.30%) and random (0.20%) condition. Following Stöttinger et al. (2018b) answers in the gradual condition were coded as “second object” as soon as the second object was mentioned. Such answers in the random condition were coded based on the indicated preference (e.g., “It could be a cat or a rabbit. But it looks more like a rabbit”). Please note that this asymmetry in the coding criteria could theoretically lead to a slight bias in favour of the gradual condition, but cannot explain if children identify the second object *later* in the gradual than in the random condition. In practice, however, it is unlikely to make a significant difference due to the very small number of cases involved (0.3% vs. 0.2%).

#### Exclusion

For seven children the data set was incomplete. Three individual sets from three different children in the gradual condition were excluded because children perseverated on a catch trial (e.g., continued to say “rabbit” in the rabbit-cat picture set although a tree was presented as a catch trial). In three individual cases (gradual: *n* = 1; random: *n* = 2), a set had to be excluded because the child refused to give an answer on several trials. One additional set had to be excluded in the random condition because the child reported the “second” object for all but one picture (i.e., picture #7).

### Go-no go task (Kerns & McInerney, 2007)

Children were instructed to react to target stimuli appearing on a 15.6inch touch screen in four separate blocks (baseline, inhibition, 2x set shifting) of 25 stimuli in each block (~ 45 s per block). In the first block (baseline) children were instructed to catch a dog that was jumping across the screen as fast as they could by touching the screen with their finger. In the second block (inhibition) an additional animal (i.e., a koala) appeared on the screen. Children were asked to “catch” the dog and to ignore the koala who “is trying to play a trick on you”. In the third and the fourth block (set shifting) rules were switched (i.e., Block 3: “Now catch the koala and ignore the dog”; Block 4: “Now catch the dog and ignore the koala”). In each of the three test blocks both characters were presented in pseudorandom order and location, with the target stimulus appearing 13 times. Before a block started, children were asked whether they knew whom they were supposed to catch. If children could not remember, rules were repeated. Six children failed to do the task (e.g., they did not want to participate or could not operate the touch screen). Following Miller et al. (2012) performance was divided into inhibition (Block 2) and set shifting (average in Block 3 & 4), where the dependent variables were (1) the difference between hits (i.e., how often a child caught the target character) minus false alarms (i.e., how often a child responded to a non-target) and (2) reaction times for hits.

### Verbal intelligence task

To measure children’s general verbal abilities, we used the subtest “Picture Naming” from the German version of the Wechsler Preschool and Primary Scale of Intelligence (WPPSI-III; Petermann, 2009). Children were presented with 26 pictures of objects and asked to name them. For each child the sum of correctly labelled objects was calculated with a maximum score of 26.

### Transparency, openness & data availability

We report how we determined our sample size, all data exclusions, all manipulations, and all measures in the study following JARS (Kazak, 2018). All the datasets are made available on OSF https://osf.io/7sqjy/. Data were analysed using SPSS, version 29. This study’s design and its analysis were not pre-registered.

## Results

### Picture morphing task

**Fig. 2:**
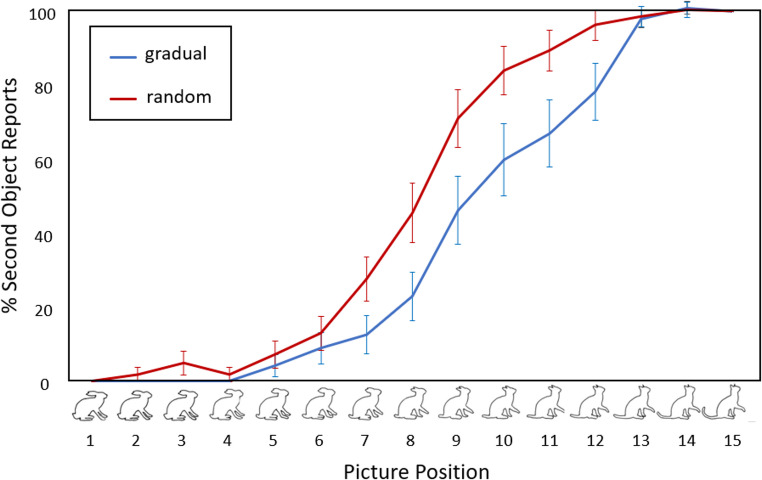
Average percentage of participants reporting the second object (y-axis) at each of the fifteen morphing stages (x-axis) averaged for all valid picture sets and age groups. Error bars reflect standard error of the mean

For analysis and display purposes, the pictures in the random condition were put back into the consecutive order of each set. Figure 2 displays the mean percentage of “second object reports” of the random and gradual condition, averaged across all children and all sets per condition. Responses in both conditions – random (red line) and gradual (blue line) – matched closely the S-curve typically found in categorical perception (Hartendorp et al., 2010; Stöttinger et al., 2016). However, responses from children in the gradual condition indicate that they recognised the second object later.

Mean picture position at which children reported the second object across all sets was submitted to a repeated-measures ANOVA with condition (gradual vs. random) as within subjects factor and age group (3- to 4-year-olds, 5-year-olds, 6-year-olds) as between subjects factor. The repeated measures ANOVA showed a significant main effect for condition, *F*(1,55) = 23.04, *p* < .001; *η²*=0.30, and age group, *F*(2,55) = 6.17, *p* < .01; *η²*=0.18. The interaction between condition and age group was not significant, *F*(2,55) = 2.49, *p* = .092; *η²*=0.08. Taken together, children identified the second object at a significantly *later* picture position in the gradual condition (at picture position *M* = 10.32 ± 1.93) compared to the random condition (*M* = 8.68 ± 1.34). Averaged across both conditions, 6-year-olds identified the second object significantly earlier (*M* = 8.86 ± 0.78) compared to 3- to 4-year-olds (*M* = 9.79 ± 1.05) and 5-year-olds (*M* = 9.80 ± 0.94), all *p*s < 0.05, Tukey’s Post-Hoc test, all *p*s < 0.05.

### Explicit memory question

Children on average remembered the initial object in one out of three sets correctly (*M* = 1.03 ± 1.11) with poorer memory in the youngest age group (*M* = 0.40 ± 0.82) compared to 5- (*M* = 1.10 ± 1.07) and 6-year-olds (*M* = 1.67 ± 1.09), *F*(1,55) = 7.72, *p* < .01, *η² =* 0.22.

### Inhibition & set shifting

In the inhibition condition children on average reached a score of 11.26 (± 2.09) out of 13, with the youngest children (*M* = 9.59 ± 2.18) reaching a significantly lower score compared to 5- (*M* = 11.45 ± 1.96) and 6-year-olds (*M* = 12.61 ± 0.61), *F*(1,52) = 13.57, *p* < .001, *η²* = 0.34; Tukey’s Post-Hoc test all *p*s < 0.05. With regards to reaction time, 5- (*M* = 973 ± 139.35ms) and 6-year-olds (*M* = 942 ± 109.81ms) reacted faster compared to 3- and 4-year-olds (*M* = 1056.52 ± 109.71ms), *F*(2,55) = 4.58, *p* < .05, *η²* = 0.14; Tukey’s Post-Hoc test, all *p*s < 0.05. A comparable performance across all three age groups was found for set shifting. Children of all age groups neither showed a significant difference in their Hit-False Alarm score (3- to 4-years: *M* = 11.50 ± 1.84, 5 years: *M* = 11.80 ± 3.02, 6 years: *M* = 13.03 ± 1.06), *F*(1,52) = 2.47, *p* = .095, *η²* = 0.09, nor in their reaction times (3- to 4-years: *M* = 1019.11 ± 105.16ms, 5 years: *M* = 947.88 ± 107.26ms, 6 years: *M* = 949.33 ± 100.43ms), *F*(2,52) = 2.89, *p* = .064, *η²* = 0.10.

### Verbal skills

On average children named 19.22 (± 3.31) of the 26 objects correctly, with a higher performance in 6-year-olds (*M* = 21.33 ± 2.87) and 5-year-olds (*M* = 19.65 ± 3.35), compared to children aged 3 and 4 (*M* = 16.90 ± 2.05), *F*(2,55) = 12.20, *p* < .01, *η²* = 0.31; Tukey’s Post-Hoc test, all *p*s < 0.05.

### Correlations

Performance in the gradual picture morphing task, responses to the explicit memory question, inhibition and verbal skills were all significantly correlated with age (see Table 1). Older children tended to be faster at identifying the second object in the picture morphing task and also showed better memory for the initial picture, higher inhibition and verbal skills. Once age was controlled for, the previously significant correlation between performance in the gradual condition and both performance in the random condition and inhibition was no longer significant. There was, however, a marginally significant negative correlation with performance in the random condition (*r* = − .25, *p* = .06): children who identified the second object earlier in the random condition tended to identify it later in the gradual condition.

**Table 1:** Correlations and [partial correlations controlling for age]

	Gradual	Memory	Random	I_score	I_RT	SS_score	SS_RT	Verbal IQ
Age	− 0.38**	0.43**	0.10	0.58**	− 0.40**	0.21	− 0.38**	0.52**
Gradual		− 0.25°	− 0.27*	− 0.31*	0.22	− 0.20	0.21	− 0.25°
Memory	[-0.10]		− 0.12	0.15	− 0.09	0.21	− 0.18	0.30*
Random	[-0.25°]	[-0.19]		− 0.02	− 0.06	0.03	− 0.18	− 0.01
I_score	[-0.14]	[-0.10]	[-0.10]		− 0.37**	0.62**	− 0.22	0.47**
I_RT	[0.03]	[0.11]	[-0.03]	[-0.21]		− 0.12	0.80**	− 0.24°
SS_Score	[-0.13]	[0.14]	[0.01]	[0.63**]	[-0.05]		0.05	0.17
SS_RT	[0.05]	[-0.03]	[0.15]	[-0.03]	[0.78**]	[0.13]		− 0.18
Verbal IQ	[-0.05]	[0.09]	[0.10]	[0.26°]	[-0.03]	[0.08]	[0.02]	

Correlations (upper half) and partial correlations (lower half) for the average picture position for which children reported the second object in the gradual condition (*Gradual*) and random (*Random*) condition, the total number of correctly remembered first objects in the gradual condition (*Memory*), inhibition (measured by Hits – False Alarms (*I-score*) as well as average reaction times for hits in Block 2 (*I_RT*)), set shifting (measured by Hits – FA – averaged over test Blocks 3 & 4 (*SS_Score*) as well as average reaction times for hits – averaged over test Blocks 3 & 4 (*SS_RT*)) and the sum of correct answers (out of 26) in the verbal IQ task (*Verbal IQ*)

** *p* < .01, * *p* < .05, ° *p* < .10, grey font = *p* ≥ .10

## Discussion

Rafetseder et al. (2021) were the first to demonstrate that children have difficulty revising their belief in response to small visual changes. This is surprising given that children and adults were exposed to the same initial picture (e.g. a rabbit) and the same gradual changes in the pictures and despite developmental research which agrees that children *are* able to adjust their beliefs when presented with contradictory evidence at an early age (Bonawitz et al., 2012; Gopnik & Bonawitz, 2015; Hagá & Olson, 2017a; Kimura & Gopnik, 2019; Köymen & Tomasello, 2018; Lane et al., 2014; Langenhoff et al., 2023; Miosga et al., 2020; Schulz et al., 2007). However, it had remained unclear whether the delayed identification observed in the picture morphing task in Rafetseder et al. (2021) – especially in the youngest children – reflected a true delay in revising their belief or whether it could instead be explained by domain general cognitive factors as well as more procedural factors such as the ambiguity of the stimulus material or an inability to follow task instructions.

To control for ambiguity, we only selected stimulus material for which “other” responses were rare in adults, ensuring that pictures were perceived categorically (Stöttinger et al., 2016). We also included a random condition to test if children perceived pictures categorically when pictures were presented in a shuffled rather than a linear order as suggested by other literature (Cheal & Rutherford, 2011; White, 2016). We hypothesised that if children only struggled with ambiguity in Rafetseder et al. (2021), they should show comparable performance in both conditions, as the composition ratios of the pictures were exactly the same. However, children in our study identified the second object significantly later in the gradual condition than in the random condition where answers closely matched the S-curve typically found in categorical perception (Fig. 2).

In addition, we improved the task instructions by including two training trials and by embedding the task within a cover story. Our analysis was based exclusively on children who had demonstrated an understanding of the task instructions. Even after controlling for these procedural factors, we replicated that children did have difficulty revising their belief in response to gradual changes in visual input, showing the robustness of this effect.

Consistent with previous studies (Kimura, 2020; Rafetseder et al., 2021), neither verbal skills nor refined measures of inhibition and cognitive flexibility explained variance in task performance beyond that already explained by age. This means that domain general abilities such as children’s ability to suppress prior beliefs or to switch between perspectives are not limiting their ability to revise their belief in a visual morphing sequence.

The fact that children identified the second object at a significantly later picture position in the gradual condition than in the random condition is the opposite of what is usually documented in healthy adult participants. It has been repeatedly shown that healthy adult participants report the second object *earlier* (i.e., when the picture still represents the first object more than the second object) in a gradually morphing context compared to a single presentation of the same picture outside that morphing context (Égrè et al., 2019.; Liaci et al. 2018; Stöttinger et al. 2016, 2018b). This suggests that healthy adults do not simply base their decision on what they see, but may instead benefit from the gradual presentation of information to generate and compare alternative interpretations against the evidence.

Together, this raises two important questions: (1) At what age do children begin to show a similar advantage from a gradual presentation compared to a random presentation of a picture series? (2) Is the local processing style typically found in younger children (Martens et al., 2014; Moses et al., 2002; Nayar et al., 2015; Poirel et al., 2008; Scherf et al., 2009) responsible for the developmental delay in belief revision in the picture morphing task?

In Experiment 2, we therefore expanded the age range from 6 to 9 years to determine when children begin to show a similar advantage for gradual over random presentation. The age range was selected based on Rafetseder et al. (2021, Experiment 2) who found that children reached adult-like performance by 9 years, and on findings from our first experiment in which we failed to find a significant interaction between age and condition until the age of 6 years. This indicates that even 6-year-olds, like 3-year-olds, were still revising their beliefs at a later position in the gradual condition compared to the random condition.

In Experiment 2 we additionally tested whether efficient identification of the second object may depend on a global processing style. Although Rafetseder et al. speculated that a local processing style can explain the late recognition of the second object in their study, we doubt that processing style can explain the variance in the performance of 3- to 6-year-old children, as most studies agree that the transition from a local to an ‘adult-like’ global bias is only happening in later childhood (between 7 and 9 years, (Martens et al., 2014; Nayar et al., 2015; Poirel et al., 2008) or even adolescence (Moses et al., 2002; Scherf et al., 2009); but see (Vinter et al., 2010). Differences in processing style may therefore only explain variance at older ages.

To test this, 6-to 9-year-old children were presented with a version of the Rey Complex Figure Test (RCF) measuring children’s processing style in addition to the gradual and random conditions of the picture morphing task. Using the Rey Complex Figure Organisational Strategy Score (RCF-OSS) by Anderson et al. (2001), children’s processing style was categorised, ranging from local, more detail-oriented to global strategies (Anderson et al., 2001; Martens et al., 2014). If a more global processing style can facilitate belief revision in the context of visual change, we expect a negative correlation between the processing level in the RCF task and the picture position at which children identify the second object. In other words, the more global the score in the RCF task is, the earlier a child should be able to recognise the second object in the gradual condition.

## Experiment 2

### Methods

#### Participants

We aimed for a similar sample size in each age group as tested in Experiment 1. Eighty-six children (44 girls) from four different age groups (6-year-olds: *n* = 15, *M*_age_= 79.34 ± 4.05 months; 7-year-olds: *N* = 23, *M*_age_= 89.91 ± 3.93; 8-year-olds: *n* = 23, *M*_age_=101.65 ± 3.35, 9-year-olds or older: *n* = 25, *M*_age_=117.77 ± 6.86) participated in this study. Each child was tested individually (á 20 min) in a quiet room. Parents gave informed written consent. Each child gave their assent prior to participation. Testing sessions were video-recorded. Children received a diploma as well as a small gift for their participation. This study followed the guidelines for developmental behavioural studies approved by the ethics committee of the University of Salzburg (EK-GZ: 42/2016).

#### Measures and procedure

Testing was done in one session with the RCF task always in between the random and the gradual condition of the picture morphing task.

#### Picture morphing task

Children were exposed to the same task and procedure as in Experiment 1 with the only exception that instructions were given without a hand puppet as we did not deem it appropriate or necessary for the age range tested in Experiment 2. In the first practice trial, 96.51% of all children recognised the second object and *all* children did so in the second practice trial, indicating that children understood the instructions of the task. A vocabulary check prior to the actual testing showed that children were familiar with the names of all objects used in the actual test. Responses in the test trials were coded by two independent raters with an inter-rater agreement of 98.50%. No child perseverated on a catch trial. On a few occasions (0.49%) children’s responses indicated both objects with a slightly smaller probability in the gradual (0.19%) compared to the random (0.30%) condition. No child reported any other object than the first or second object. Despite the successful vocabulary check, one individual set of one child in the random condition had to be excluded because the child failed to name the first object in the actual task (i.e., saw).

The order of the picture morphing task condition (gradual or random first), the assignment of the sets (to gradual or to random) and the exact order of the sets (order 1: Set1 - Set2 - Set3 or order 2: Set3 - Set2 - Set1) was counterbalanced and varied between participants using a Latin Square Design.

#### Ray Complex Figure Task (RCF)

**Fig. 3:**
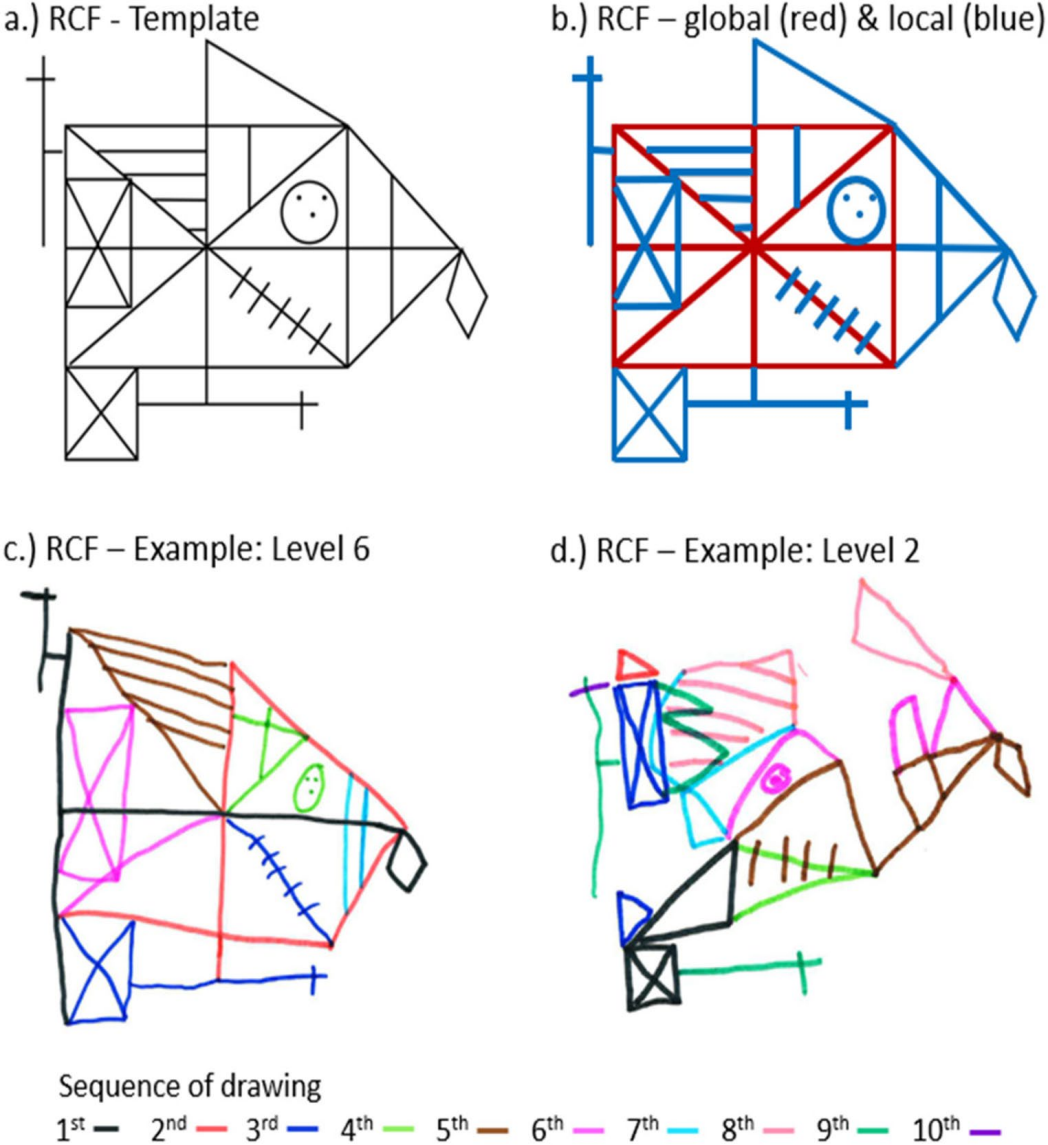
Rey complex figure. Note: **a** Rey Complex Figure presented to children. **b** Colour coded for a better understanding of the separate elements. Red = global elements. Blue = local elements. **c** Example Level 6. **d** Example Level 2. The colours in c & d reflect the sequence of the order in which the elements have been drawn

We used a version of the Rey Complex Figure task (RCF, Fig. 3a) that consisted of global elements (red) and local elements (blue, Fig. 3b; see also Table SI_1 in Supplemental Materials). Half of the local elements were aligned along the horizontal centre line, and half were aligned along the vertical centre line. The RCF was printed on a white DIN A4 sheet and placed in front of the child. The child was asked to copy the figure to the best of their ability on a separate sheet of paper. Every 30 s the child was given a different pencil-colour (Fig. 3c and 3d). This allowed for recording the order in which each element of the figure was drawn. The child was told that this is done to create a really colourful picture. Children were given a maximum of 5 min (i.e., 10 different pens) to complete the copy.

##### Coding

Each element of the figure was rated by two independent coders whether (1) the element was present, (2) when it was drawn and (3) whether or not it was complete. Children were classified on a 7-point RCF-OSS scale described in Anderson et al. (2001) with higher scores indicating a more global processing style. Inter-rater agreement (Spearman’s *r* = .85, *p* < .001) was comparable with Anderson et al. (2001) which was between 0.85 and 0.92. In cases of disagreement the higher level was assigned. Both raters were blind as to the performance of the child in the picture morphing task (for details see Supplemental Materials).

## Results

### Picture morphing task

Like in Experiment 1, the mean picture position at which children reported the second object was submitted to a repeated-measures ANOVA with condition (gradual vs. random) as within subjects factor and age group (6-year-olds, 7-year-olds, 8-year-olds, 9-year-olds or older) as between subjects factor. Children, on average, did not demonstrate a benefit for the gradual presentation. They reported the second object at a similar picture position across both conditions: *M*_*Gradual*_ = 9.01 ± 1.46; *M*_*Random*_ = 9.00 ± 1.09; *F*(1,82) = 0.06, *p* > .80, *η²* = 0.001] with an only marginally significant main effect for age group [*F*(3,82) = 2.32, *p* = .081; *η²* = 0.08] and no significant interaction between condition x age group [*F*(3,82) = 1.74, *p* > .15, *η²* = 0.06].

### Explicit memory question

Children on average remembered the initial object in two out of three sets correctly (*M* = 2.37 ± 1.87). Older children remembered more initial objects than younger children (Table 3).

### RCF task

All children made an attempt to draw the figure (i.e., > Level 1). Only one child reached level 6 (Fig. 3c) and no child reached level 7. The majority of 6- and 7-year-old children (~ 67%) used poor organisation strategies (i.e., Level 1–3; see Fig. 3d for an example of Level 2). In contrast, older children (aged 8 to 9) used advanced strategies more frequently (~ 65%), see Table 2. Our data closely resemble the data reported by Anderson et al. (2001).

**Table 2:** Number of children at each organizational level separate for age group

	Level 1	Level 2	Level 3	Level 4	Level 5	Level 6	Level 7
6 years(*N* = 15)	0	2	8	3	2	0	0
7 years (*N* = 23)	0	1	13	5	4	0	0
8 years (*N* = 23)	0	1	7	6	9	0	0
≥ 9 years (*N* = 25)	0	0	9	6	9	1	0

### Correlations

Performance in the gradual condition and the RCF task improved significantly with age. Performance in the RCF task remained significantly correlated with performance in the gradual condition, even after controlling for age. Children with a more global processing style identified the second object at an earlier picture position than those with a more local processing style (Table 3). Children who had a better memory of the first object were quicker to identify the second object. This correlation remained marginally significant after controlling for age (*r* = − .21, *p* = .06).

**Table 3:** Correlations and [partial correlations controlling for age]

	RCF Task	Gradual	Random	Memory
Age	0.31^**^	− 0.25^*^	0.03	0.24^*^
RCF Task		− 0.28^**^	0.13	0.04
Gradual	**[-0.22** ^*^ **]**		**− 0.18°**	**− 0.25** ^*^
Random	**[0.12]**	**[-0.18]**		**− 0.03**
Memory	**[-0.03]**	**[-0.21°]**	**[-0.04]**	

** *p* < .01, * *p* < .05, ° *p* < .10, grey font = *p* ≥ .10

### Analysis of the developmental trajectory

For this analysis we collapsed the data from Experiments 1 and 2, and compared them with the performance of adult participants tested in Stöttinger et al. (2018b). In this study, healthy adults were presented with the gradual and random conditions, differing only in one set of pictures (Frog-> Person). We removed that picture set and restricted the analysis to only those five sets that were presented to all participants across the three experiments. This allowed for a direct comparison of children’s performance and to investigate at what age they reached adult-level performance.

**Fig. 4:**
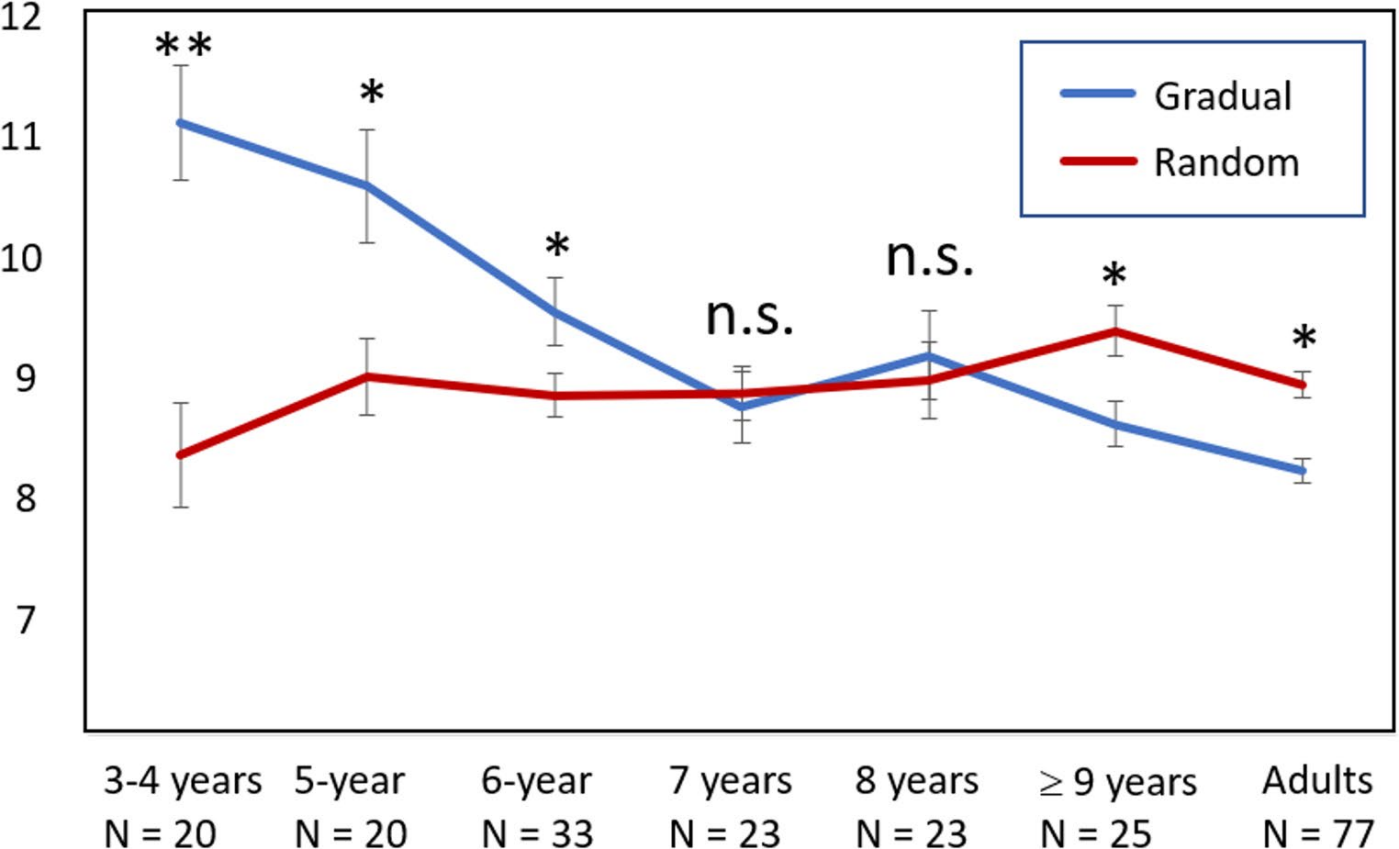
Average number of picture positions at which each age group identified the second object collapsed across Experiment 1 and 2, and additional data from Stöttinger et al. (2018b). Note. Sets were restricted to only those that were presented to all participants. Error bars reflect standard error of the mean.

The mean picture position at which participants reported the second object was submitted to a repeated-measures ANOVA with condition (gradual vs. random) as within subjects factor and age group (3- to 4-year-olds, 5-year-olds, 6-year-olds, 7-year-olds, 8-year-olds, 9-years-and-older, healthy adults tested in Stöttinger et al. 2018b) as between subject factor (Fig. 4). This analysis revealed a significant main effect for condition, *F*(1,214) = 11.81, *p* < .01; *η²*=0.05, a significant main effect for age group, *F*(6,214) = 8.58, *p* < .001; *η²* =0.19, and a significant interaction between condition and age group, *F*(6,216) = 10.64, *p* < .001; *η²* =0.23.

Two separate univariate ANOVAs for condition revealed a significant main effect for age group for the gradual condition only, *F*(6,214) = 15.72, *p* < .001; *η²* =0.31. Three- to four-year-olds (*M =* 11.07 ± 2.12), 5-year-olds (*M =* 10.54 ± 2.12) and 6-year-olds (*M =* 9.5 ± 1.61) identified the second object at a significantly later picture position compared to adults (*M =* 8.19, ± 0.90; Turkey’s Post-Hoc tests, all *p*s < 0.05). No significant difference was found between adults and 7-year-olds (*M =* 8.71 ± 1.43), 8-year-olds (*M =* 9.14 ± 1.76) and children 9 years and older (*M =* 8.57 ± 0.83). The same analysis calculated for the average picture position in the random condition did not find a significant age effect, *F*(6,214) = 1.36, *p* > .20; *η²* =0.04; Turkey’s Post-Hoc Tests all *p*s > 0.05.

In a second step we compared the average picture position at which participants identified the second object in the gradual and random condition, separately for each age group, using paired sample t-tests. Three- to four-year-olds, *t*(19) = 3.74, *p* = .001, *q*^1^ = 0.006, *d* = 0.84, 5-year-olds, *t*(19) = 2.47, *p* = .01, *q* = 0.02, *d* = 0.55, and 6-year-olds, *t*(32) = 2.10, *p* = .02, *q* = 0.03, *d* = 0.37, reported the second object at a significant *later* picture position in the gradual compared to the random condition. No significant difference was found for 7-year-olds, *t*(22) = 0.29, *p* = .39, *d* = 0.06, and 8-year-olds, *t*(22) = 0.36, *p* = .36, *d* = 0.07. Only children 9 years and older identified the second object *earlier* in the gradual compared to the random condition *t*(24) = -3.24, *p* = .002, *q* = 0.006, *d* = 0.65, resembling the pattern found in adults (e.g., Stöttinger et al. (2018b)).

## Discussion

In Experiment 2, we examined the point at which children begin to exhibit the same advantage of gradual over random presentation that has previously been observed in adults (Egre et al., 2019; Liaci et al. 2018; Stöttinger et al. 2016, 2018b). Unlike the 3- to 6-year-old children in Experiment 1, the 6- to 9-year-old children in Experiment 2 identified the second object at a *similar picture position* in the gradual and the random condition, with no significant interaction with age. To gain deeper insight into the developmental progression of belief revision in the visual context, we conducted an additional analysis using three data sets. We combined data from Experiment 1 and Experiment 2, and compared them with adults tested in Stöttinger et al. (2018b). We found that only 9-year-old children showed the benefit for gradual presentation, while younger children either did not show this benefit (7- to 8-year- olds) or were even hindered by the gradual presentation (3- to 6-year-olds).

Using the RCF task we found further that, children who scored higher identified the second object earlier in the gradual condition compared to children who scored lower. While this provides evidence that a more global processing style is linked with recognition of the second object in 6-to 9-year-old children, the factors underlying the difficulty of 3- to 6-year-olds in updating their beliefs in response to gradual visual changes remain unclear.

In order to efficiently revise a belief in the context of the picture morphing task, participants must recognise that some interpretations are more plausible than others and that any new interpretation must be consistent with the current stimulus. This process requires the ability to generate mental templates of possible objects and map them onto the stimulus in order to determine whether they provide a viable match — an ability known as mental imagery. According to Kosslyn and colleagues (Kosslyn, 1996; Kosslyn et al., 2006) mental imagery relies on several sub-processes including (a) the creation of an image within a short-term ‘depictive buffer’, either by reactivating perceptual information about an object or by combining different elements to create a new image; (b) the inspection of this image by selectively focusing on a particular feature of the image; (c) the maintenance of an image in memory, together with (d) a transformation of the image, such as the mental rotation of imagined objects. Given that these sub-processes depend on other cognitive abilities the most substantial development occurs between 8 and 14 years of age (see Burnett Heyes et al., 2013 for a review).

In Experiment 3, we therefore eliminated the need to create a mental image to search for alternative interpretations. To achieve this, children were asked to sort test cards either into a box with the picture of the first object (e.g. rabbit) on the front, or into a box with the picture of the second object (e.g. cat) on the front. By printing the objects on the boxes, there was no need to use mental imagery to generate new hypotheses about what the object might become. Children could simply hold the card up to the picture of each box and decide whether it looked more like the first or the second object.

Performance on this manual task was then compared with performance on the original computerised gradual version of the task. We decided to focus on 5- to 6-year-olds as they were the ones who were negatively impacted by the gradual presentation in Experiment 1 and yet had the best chance of benefiting from a visual presentation of the second object. We hypothesised that if 5- to 6-year-olds struggle to revise their beliefs in the picture morphing task due to an inability to use mental imagery to form new hypotheses, eliminating the need for exploration should improve their performance in the manual version of the gradual condition compared to the original task.

## Experiment 3

### Participants

A total of 47 5- to 6-year-old children (23 girls; *M*_age_= 67.13 ± 4.20 months; ranging between 60 and 74 months) participated in this experiment. One additional child was tested but had to be excluded due to non-compliance. Children were recruited from three different nurseries. Each child was tested individually in one session (á 15 min) in a quiet room. Parents gave informed written consent to test and video-record their child and each child gave their assent prior to participation. Children received a diploma as well as a small gift for their participation. This study followed the guidelines for developmental behavioural studies approved by the ethics committee of the University of Salzburg (EK-GZ: 42/2016).

### Picture morphing task

Children were exposed to four picture sets (i.e., Violin-> Pear, Shark-> Plane, Spider-> Sun, Rabbit-> Cat; https://osf.io/qi2vg/), half of which were presented on the computer (gradual - original). Like in Experiment 1 instructions were given with a hand puppet, thus replicating the procedure of Experiment 1. For the gradual - manual condition the pictures of the other half were printed on 8 cm x 8 cm white cards, and the children were asked to sort the cards into one of two cardboard boxes (length: 16.5 cm x height: 13 cm) - either the box with the first object of the picture series on the front, or the box with the second object of the picture series on the front. A third box was decorated with coloured feathers and was presented to the children as a ‘crazy box’. In this box they were asked to place any pictures that did not match either the first or the second object (i.e., resembling catch trials).

A vocabulary check prior to the actual testing guaranteed that children were familiar with the names of all objects used in the actual test. In the first practice trial, 80.85% of all children recognised the second object and *all* children did so in the second practice trial, indicating that children understood the instructions of the task. Responses in the gradual-original condition were coded by two independent raters with an inter-rater agreement of 98.78%. In only very rare occasions (0.03%) did children’s responses indicate both objects at once. Children did not report any other objects than the first or second object. In six individual sets of the manual condition children inserted one of the test cards in the “crazy box”. In this case the “crazy box” was coded as “second object”, in line with how “other” reports were coded as “second object” in the original condition in Experiment 1 and 2.

One picture set was excluded from the original condition because the child was randomly labelling each picture. Two sets had to be excluded in the gradual-manual condition because the picture sets were presented in the wrong order and data was thus not comparable to the results of Experiment 1 and 2. No child perseverated on a catch trial.

Following the procedure of Experiment 1 and Experiment 2, the dependent variable was the picture position at which children indicated the second object. The order of the picture morphing task condition (gradual-original or gradual-manual first), the assignment of the sets (to gradual-original or to gradual-manual) and the exact order of the sets (order 1: Set1 - Set2 or order 2: Set2 - Set1) was counterbalanced and varied between participants using a Latin Square Design.

## Results

**Fig. 5:**
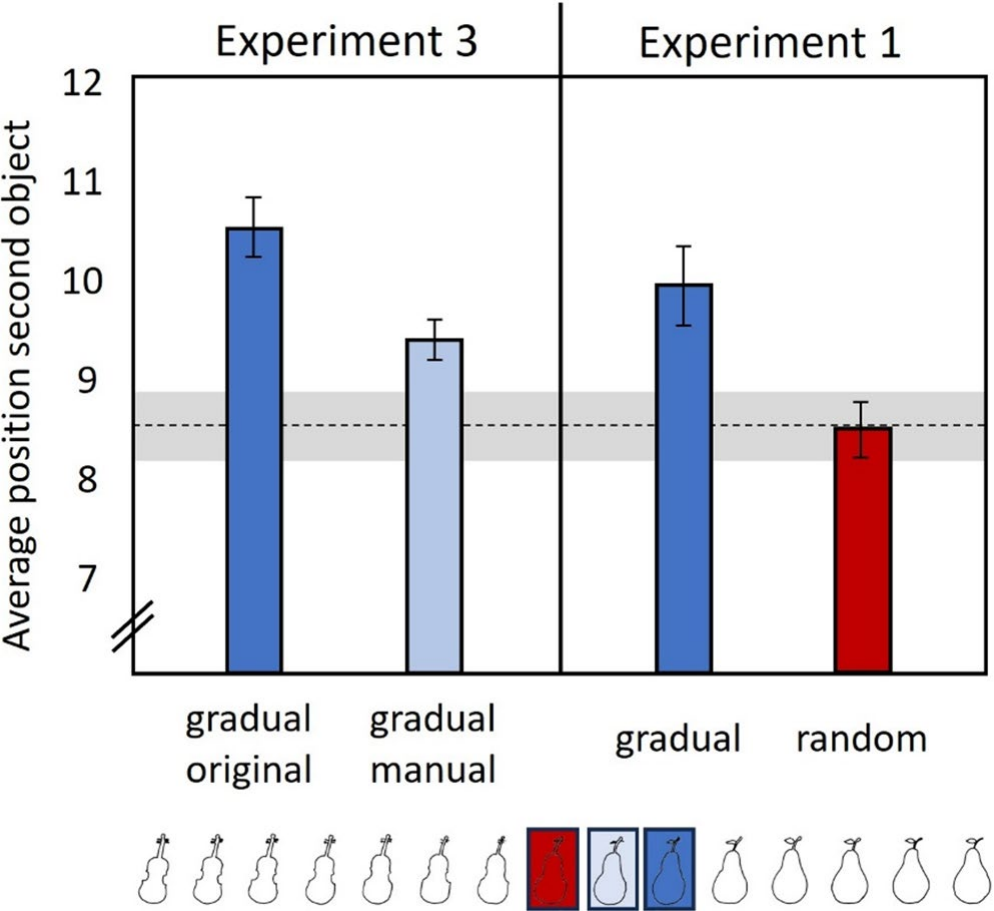
Average number of picture positions at which participants identified the second object in Experiment 3 (left), Experiment 1 (right). Note. The dotted line displays the average performance in the gradual condition of healthy adults in Stöttinger et al. (2018b) together with the 95% confidence interval. Analysis was restricted to only those four sets that were presented to all participants across the experiments. For the analysis of Experiment 1 only 5- to 6-year-old children were included. Error bars reflect standard error of the mean

Children on average identified the second object significantly earlier in the gradual-manual condition (*M*_*Manual*_ = 9.36 ± 1.40) compared to the gradual-original condition (*M*_*Original*_ = 10.48 ± 2.05), *F*(1,45) = 8.54, *p* < .05, *η²* = 0.16 (see Fig. 5).

### Exploratory analysis

For an exploratory purpose we compared the performance of children in Experiment 3 with the performance of children in Experiment 1. This analysis was restricted to 5- to 6-year-olds and only to those sets used in both experiments. Performance in the gradual-original condition of Experiment 3 resembled performance in the gradual condition of Experiment 1 (*M*_*Gradual*_ = 9.91 ± 2.45), *t*(46) = 1.90, *p* > .05, *d* = 0.28. Children again identified the second object significantly later in the gradual-original condition in Experiment 3 compared to the Random condition in Experiment 1 (*t*(46) = 6.71, *p* < .01, *d* = 0.98). Interestingly, despite removing the need for visual imagery in the gradual-manual condition in Experiment 3, children still identified the second object significantly later in this condition compared to children in the random condition in Experiment 1, (*M*_*Random*_ = 8.47 ± 1.72), *t*(45) = 4.30, *p* < .01, *d* = 0.63 (Fig. 5).

## Discussion

Removing the need to use mental imagery to explore for potential alternative interpretations led to a significant improvement in Experiment 3. By giving children a visual choice between the first and the second interpretation of the object we eliminated the need for children to maintain a mental representation of the first object as well as the need to generate a mental image for a potential second object. Instead of relying on memory and internal visualization, children could simply refer to the external images. This made it easier to detect when the transformation had progressed enough to switch their response. In essence, the task may have shifted from a more cognitive process (mentally maintaining and visualising an evolving image) to a more perceptual one (matching the changing image to an already visible reference). In this condition, children identified the second object significantly earlier than in the original version of this task. Children however still seemed to be hindered by the gradual presentation as they needed more pictures to identify the second object compared to children in the random condition in Experiment 1 who did not have a visual reference point. We will explore potential explanations of this difference in the General Discussion.

## General discussion

The ability to revise beliefs in response to new evidence is a fundamental cognitive skill that underpins learning and adaptation in uncertain environments. The literature agrees that children, even as young as 4 years old are able to revise their beliefs in response to contradictory evidence from the environment (Bonawitz et al., 2012; Gopnik & Bonawitz, 2015; Hagá & Olson, 2017a; Kimura & Gopnik, 2019; Köymen & Tomasello, 2018; Lane et al., 2014; Langenhoff et al., 2023; Lucas et al., 2014; Miosga et al., 2020; Schulz et al., 2007). However, Rafetseder et al. (2021) showed that children up to the age of 9 revise their belief in response to gradual visual information significantly later than adult participants, despite being exposed to the same initial information and the same contradictory evidence. We replicated this developmental lag in three experiments and explored potential explanations.

In Experiment 1, we investigated whether the effects could be replicated when potential procedural and domain general cognitive factors were controlled for. We still found that children needed more perceptual counter-evidence before identifying the second object in the gradual condition while showing an adult-like categorical perception when the same images were presented in a random order. Neither inhibition, cognitive flexibility, nor verbal skills explained variance beyond age. This suggests that neither procedural factors like the ambiguity of the stimulus material nor domain-general executive abilities are the limiting factor in this task. These results clearly demonstrate the robustness of Rafetseder et al.‘s (2021) findings.

The results of Experiment 2 suggest that from the age of 6 years, children, who adopt a more global (holistic) processing style (i.e., perceiving the overall structure or gestalt of an image before focusing on specific details), are more efficient to revise their beliefs. When the need for mental imagery was removed in Experiment 3 by making both interpretations visually available, children aged 5 to 6 years performed better, although not to the level observed in the random condition of Experiment 1 and not to the level observed in adult participants tested in Stöttinger et al. (2018b).

Analysing the developmental trajectory across several datasets revealed an age-related advantage for the gradual over the random condition. While 3- to 6-year-old children were hindered by gradual changes, 7- to 8-year-olds did not show the typical advantage for gradual over random presentation observed in healthy adult participants (Égré et al., 2019, Liaci et al. 2018; Stöttinger et al. 2016, 2018b). Only children aged 9 and over – like adults – were able to use smooth transitions to detect the second object earlier (Experiment 2). Overall, the results of our three experiments suggest that children - unlike adults – face challenges when they process gradual changes in visual stimuli and that these challenges are different for children of different ages.

In healthy adults, it has been repeatedly reported that they benefit from a gradual over random presentation (Égrè et al., 2019; Liaci et al. 2018; Stöttinger et al. 2016, 2018b). Importantly, this indicates that their decisions are not solely stimulus-driven (i.e. representing the actual composition of the picture), but also involve the active exploration of alternative interpretations (e.g. ‘I know it’s a rabbit, but what else could it be?‘). Data reported in other studies using the same task supports this hypothesis. For example, Stöttinger et al. (2018a; see Fig. 2b) showed a systematic increase in reaction times two pictures before adults reported the second object. Meanwhile, Wainstein et al. (2025; see Fig. 1C) reported an increase in pupil diameter before participants switched to a new interpretation. Since longer reaction times (Kiani et al., 2014) and task-evoked changes in pupil diameter (Greg et al., 2015) are associated with reduced certainty and uncertainty promotes exploratory behaviour (Bold et al., 2019), this pattern plausibly suggests an active search for alternative perceptual hypotheses in healthy adult participants before the switch.

The absence of a gradual-over-random advantage in 7- to 8-year-olds may indicate that they do not actively search for alternative interpretations and that their decision is more stimulus-driven, potentially due to a more local processing style. Consequently, their decisions depend primarily on the actual composition ratios of the images, which are identical across gradual and random conditions.

However, this explanation does not hold for 3- to 6-year-olds who are hindered by the gradual presentation. That is, they identified the second object *later* as justified by the objective perceptual input (i.e. random condition). Even if they could compare the changes with a visual representation of the target objects in Experiment 1, they still needed more pictures to identify the second object compared to children in the random condition in Experiment 1.

At that point we can only speculate what may have caused this delay. One possibility is that it reflects age-related differences in prediction error sensitivity, which are reduced by presenting the target objects visually (i.e. gradual manual condition of Experiment 3). According to predictive coding theory (Friston & Kiebel, 2009; Rao & Ballard, 1999), the brain maintains a probabilistic model of the world (i.e., ‘priors’). These priors are then used to predict sensory input. When sensory input differs from these predictions, a prediction error is produced which serves as a signal to revise the initial beliefs (see Rapaport et al., 2023, for a review). Using a standard passive auditory oddball paradigm, Rapaport et al. (2023) showed that the precision of these priors matures as children gain experience of the world. In their task, 4- and 6-year-old children were presented with sequences of highly probable tones, interspersed randomly with tones of a low probability (i.e., oddballs). Recording the children’s neural responses to oddballs enabled the researchers to infer the precision of the children’s priors. The researchers found that 6-year-olds exhibited a greater prediction error in response to oddballs than 4-year-olds did. This indicates that 4-year-olds have less precise predictions and consequently exhibit a weaker response to oddballs.

It is possible that younger children did not exhibit a prediction error response to slightly atypical animals (e.g., a rabbit with small ears and a long tail), as these stimuli may still have been accommodated within the existing category representation under the initial prior (i.e. it is still a rabbit, albeit with oddly shaped ears and a very long tail). In Experiment 3 (gradual manual condition), the second object was visually presented, which may have reduced younger children’s prediction error by enabling a direct visual comparison between the stimuli. Similarly, the broader and more flexible category boundaries observed in kindergarten children (Abecassis et al., 2001) may also explain why 3- to 6-year-olds in our study were hindered by the gradual presentation. They may treat each successive image as another acceptable instance of the initial category rather than as disconfirming evidence. When a rabbit gradually morphs into a duck, each intermediate image still shares substantial perceptual overlap with the initial representation. Three- to 6-year-olds may therefore interpret each slightly altered image as “another kind of rabbit,” effectively broadening the boundaries of the original category instead of questioning its validity. In this account, children are not failing to detect change per se; rather, they assimilate incremental deviations into an increasingly tolerant category representation. This interpretation is consistent with research suggesting that young children often exhibit broader or more flexible category boundaries and may accept atypical exemplars as members of a familiar category, especially when matching labels contribute to similarity among compared entities (Sloutsky & Fisher, 2004). It also aligns with evidence that children rely heavily on perceptual similarity when making category judgments, especially until the age of 6 years (Badger & Shapiro, 2012).

From this perspective, younger children may be engaging in belief updating rather than belief revision. Instead of abandoning the “rabbit” hypothesis and generating an alternative (e.g., “duck”), they update their representation of what counts as a rabbit in light of each new exemplar. The repeated exposure to slightly altered exemplars in the gradual condition may have therefore progressively expanded the child’s internal representation of the initial category, delaying the point at which a competing interpretation becomes more plausible. In Experiment 3 (gradual manual condition), providing a representation of the target object may have triggered belief revision earlier, rather than simple belief updating through boundary extension.

As we cannot draw any conclusions from our current data, future studies should investigate whether less precise priors and/or more flexible category boundaries contribute to the weaker sensitivity to changes observed in 3- to 6-year-olds, and thus to their delayed recognition of the second object. In healthy adults, increases in reaction times (Stöttinger et al. 2018a) and pupil diameter (Wainstein et al., 2025) prior to a perceptual switch have been linked to uncertainty and the exploration of alternative perceptual hypotheses. If children fail to show comparable pre-switch dynamics, this may indicate greater certainty in their initial interpretation and reduced exploration of alternatives. Accordingly, reaction time analyses and pupillometry may provide a promising avenue for further examining developmental differences in perceptual belief revision.

## Conclusion

The present findings demonstrate that children’s difficulty in detecting and responding to gradual change is robust and persists until approximately 9 years of age. Across three experiments, this robust delay could not be attributed to domain-general executive limitations or procedural-dependent factors. Instead, performance improved when children had a tendency towards a global processing style and when task demands reduced reliance on mental imagery. Further research should examine whether imprecise predictive priors or broader, more flexible category boundaries explain why especially younger children’s belief revision is affected by gradual change.

## Supplementary Information

Supplementary Material 1.

## Data Availability

Data and materials supporting this study can be found on https://osf.io/7sqjy/ once the paper has been accepted for publication; picture sets as well as normative data for each set can be found on https://osf.io/qi2vg/.

## Footnotes

1 q = Corrected p-values using the Benjamini-Hochberg (BH) procedure. See Glickman et al. (2014) for more details.
