# Supplementary material for: Children require more evidence to revise beliefs under gradual perceptual change
Source: Psychol Res. 2026 Jun 3;90(3):110. doi: 10.1007/s00426-026-02319-0 (PMC13234064; doi:10.1007/s00426-026-02319-0)
Supplement: Supplementary file 1 — Supplementary Material 1. [file 426_2026_2319_MOESM1_ESM.docx]

**Supplemental Materials**

Belief revision is thought to recruit several domain-general cognitive processes.

Individuals must form and evaluate their beliefs about the world. These beliefs are compared to the events in the real world. Based on this comparison, the learner must then suppress any prior beliefs that are not supported by the evidence and flexibly change their beliefs in response to contradictory evidence (Colantonio et al., 2024). However, children typically have limited inhibitory control and cognitive flexibility, which develop gradually from infancy (e.g., Diamond, 1985) through adolescence (e.g., Carlson & Moses, 2001). Surprisingly, tasks like the Day-Night task (Gerstadt et al., 1994) and the Simon task (Davidson et al., 2006), which measure inhibition, did not explain additional variance in the picture morphing task beyond age in Rafetseder et al. (2021). This aligns with (Kimura, 2020, [Experiment 1 & 2](https://escholarship.org/content/qt5x49v8dk/qt5x49v8dk_noSplash_7e5bb8fdd6047f3db8b1dfe9abf207b8.pdf)) who did not find a predictive power of the Day-Night task in their belief revision task and suggests that failures in suppressing natural associations or managing conflicting spatial information do not account for the delayed identification in the picture morphing task.

Despite these findings, we included a version of the Go-NoGo task (Kerns & McInerney, 2007) in our study to measure both inhibition and cognitive flexibility. Children had to respond to a target stimulus (e.g., a dog) while ignoring other stimuli (e.g., a koala). Inhibition was measured not only by hits but also by omission errors, commission errors and reaction times. It is possible that these refined measures allow to detect more subtle effects of inhibition on task performance. We hypothesised that, if inhibition demands of the picture morphing are beyond the capacity of young children, an earlier report of the second object should correlate with a higher inhibition score after controlling for age.

In addition, set-shifting was measured in two additional blocks, in which participants had to switch rules (“Catch the koala and ignore the dog.”; “Catch the dog and ignore the koala.”). Although young children may struggle with rule shifts, Kimura's (2020) Experiments 1 and 2 suggest that this is unlikely. In their study children’s set-shifting performance in the Dimensional Change Card Sort (DCCS) task was not related to performance on the belief revision task. However, the DCCS may not capture set-shifting effects as effectively as the more complex Go-NoGo task. We hypothesised that if the developmental lag observed in Rafetseder et al. (2021) was due to limitations in cognitive flexibility, then an earlier identification of the second object should be associated with a higher set-shifting score, even after controlling for age.

Finally, children’s limited verbal skills may have further prevented them from naming the object, either because they did not know the label of the transformed object, or because limited verbal skills hindered their ability to grasp counterintuitive concepts (Gopnik & Astington, 1988). To assess the effect of general verbal ability, we used a subtest of the Wechsler Preschool and Primary Scale of Intelligence (WPPSI-III; Petermann, 2009). We hypothesised that, if the delay in reporting the second object was due to limited verbal skills, earlier reporting of the second object should correlate with a higher verbal score, even after controlling for age.

Gradual Picture Morphing Task Instructions:

Testing was done by two research assistants. Two weeks prior to testing they went to the kindergarten to introduce themselves. They also brought along “Freddy the magic sheep” who performed a magic trick. This was done to break the ice and to introduce the children to the idea that Freddy had actual “magic power”.

At the day of testing children were asked whether they still remember Freddy and if they are curious about his other magic tricks. Two paper slips (42 x 6.5 cm) depicting images of picture sets were used. None of the pictures used for introduction were used later in the test. The first paper slip contained all fifteen images of the bear-pig picture set (Stöttinger et al., 2016). Children were presented with the first object (i.e., bear), while the remaining fourteen pictures of the set were still hidden inside a 10 x 50 cm golden paper cover. They were asked if they knew what the object was. They were then informed that Freddy will now transform the object into a completely different object. After Freddy said his spell the paper clip was slowly pulled out of the cover while children were asked repeatedly whether they already knew what the bear will turn into. The majority of children (i.e., 75%) were able to identify the second object (i.e., pig) after the paper slip was pulled out completely. The answer was confirmed by the instructor at the end: ”Exactly, Freddy has turned the bear step by step into a pig”. In cases where participants were unable to identify the second object, the answer was given to the children. They were then asked if they wanted to see another trick. A second paper slip was used containing only ten pictures (i.e., nine pictures of the fly-fir tree picture set (Stöttinger et al, 2016) together with an additional picture that served as “catch trial”). This time one picture at a time was pulled out of the cover and children were asked to report for each picture what they saw. After three consecutive “fly-pictures” had been presented, the catch trial (i.e., a picture of a mushroom) appeared. Children were told that Freddy hid something else there and they should keep looking out for the second object (i.e., “Let’s see what the fly will turn into”). As soon as a child identified the second object the instructor replied “Yes, it is possible. Let’s see if this is correct” and continued the procedure. All children were able to identify the second object at the end showing that all children understood the purpose of the task. After completion, the instructor repeated that Freddy had turned the fly slowly into a fir-tree. Children were then asked whether they wanted to see more things Freddy had transformed, but this time on the computer.

**Experiment 1:**

*Gradual-Repeat condition*

One picture set in the gradual condition was repeated to test whether children would identify the second object earlier if they knew what they were looking for. As three children did not complete this task, only 55 children were included in this analysis. Mean picture positions at which children reported the second object were submitted to a repeated-measures ANOVA with condition (gradual vs. gradual-repeat) as within subjects factor and age group (3- to 4-year-olds, 5-year-olds, 6-year-olds) as between subjects factor. This analysis failed to show a significant main effect for condition, *F*(1,52) = 3.31, *p* = .075; *η²* = .06. Children identified the second object at about the same picture position in the repeat condition (*M =* 9.20 ± 2.44) compared to the first presentation of the same picture set (*M =* 9.76 ± 2.41). Performance averaged across both conditions significantly improved with age, *F*(1,52) = 5.63, *p* < .01; *η²* = .18 with 6-year-olds identifying the second object significantly earlier than 3- to 4-year-olds; Tukey's Post-Hoc test, *p* < .05. There was no significant interaction between condition and age group, *F*(1,52) = .45, *p* > .60, *η²* = .02 (see Figure SI_1).


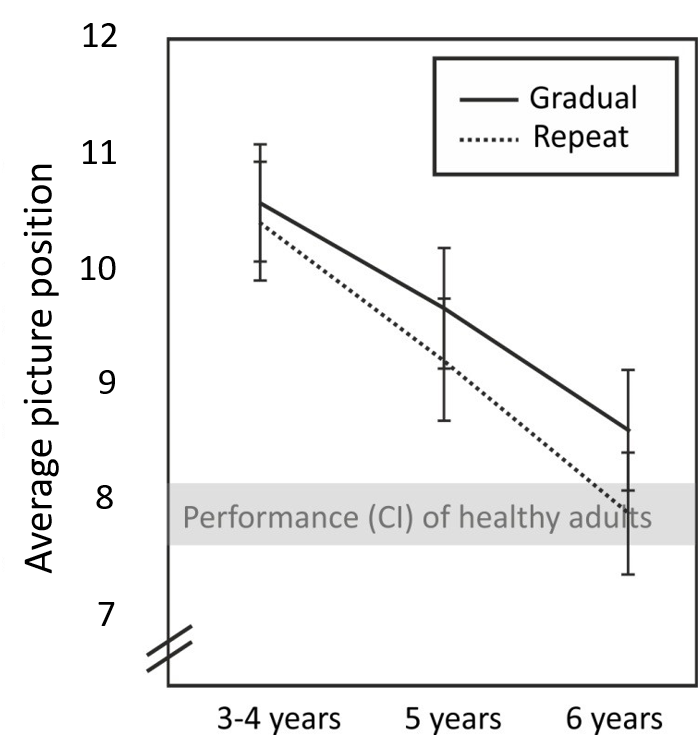


Figure SI_1: Average picture position at which participants identified the second object displayed for the picture set that was presented twice. The solid line displays the average performance for the first presentation, the dotted line displays the average performance for the second presentation. The error bars represent the standard errors of the mean. The grey horizontal bar represents the 95% confidence interval for the performance of healthy individuals in Stöttinger et al. (2018) for this set in the gradual condition.

Table SI_1 RCF Task – Overview

| Configural elements | |
| --- | --- |
| Central Lines (Z1 & Z2) | 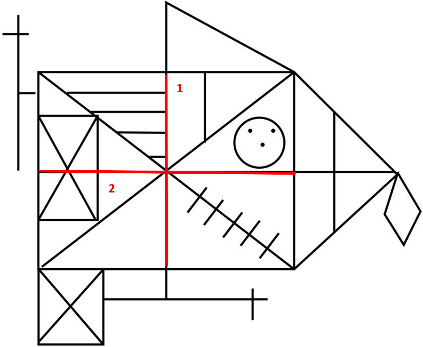 |
| Rectangle  R1, R2, R3, R4 | 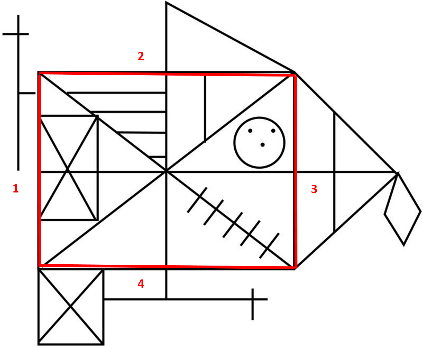 |
| Diagonales | 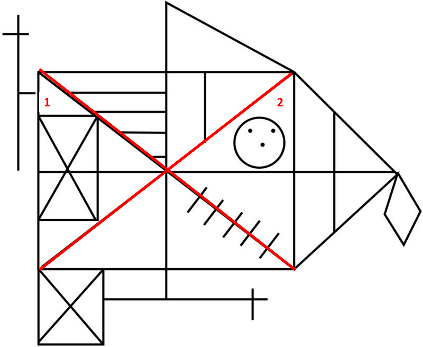 |
| **Outside local elements – vertically aligned** | |
| AV_1 | 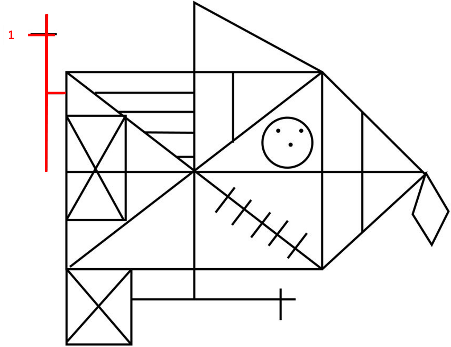 |
| AV_2 | 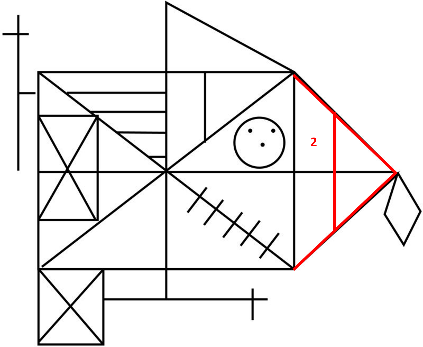 |
| AV_3 | 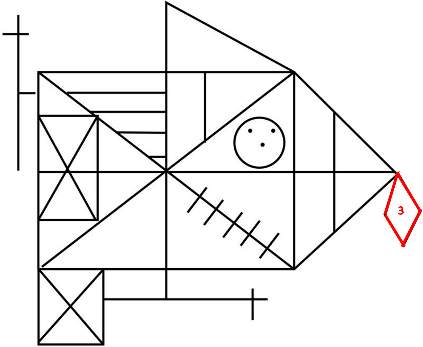 |
| **Outside local elements – horizontally aligned** | |
| AH_1 | 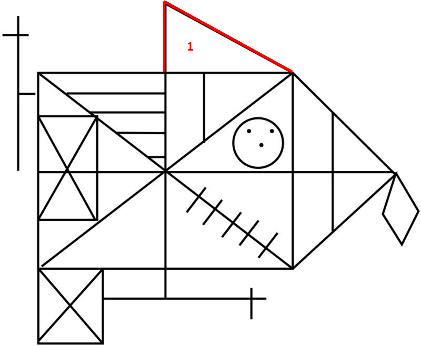 |
| AH_2 | 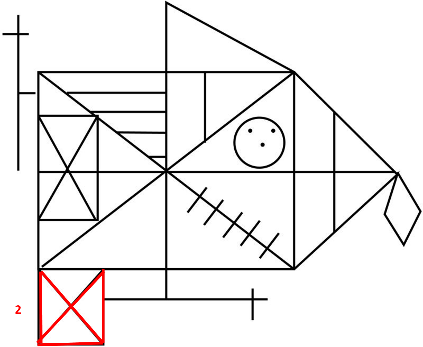 |
| AH_3 | 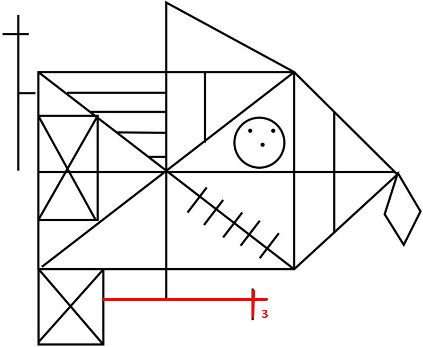 |
| **Inside local elements – vertically aligned** | |
| IV_1 | 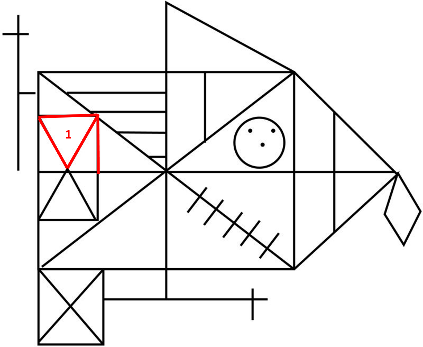 |
| IV_2 | 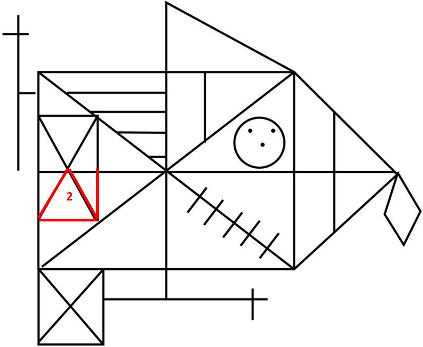 |
| IV_3 | 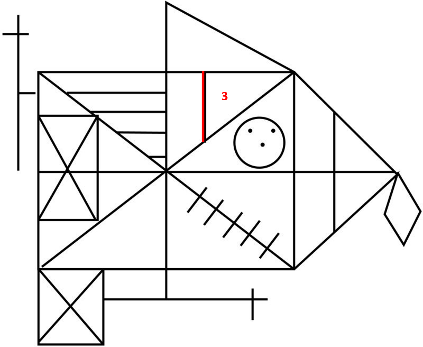 |
| **Inside local elements – horizontally aligned** | |
| IH_1 | 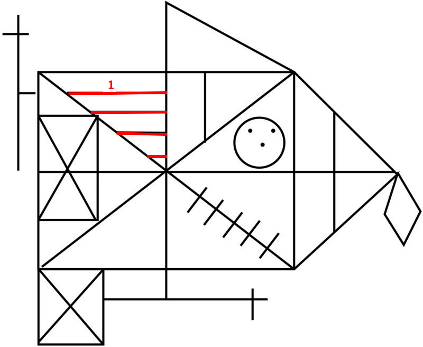 |
| IH_2 | 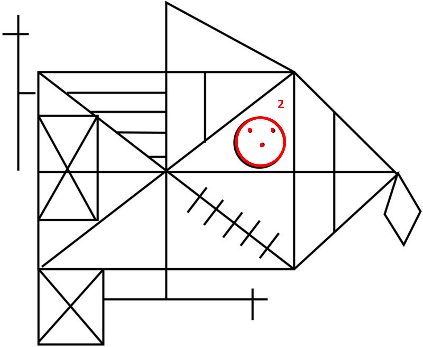 |
| IH_3 | 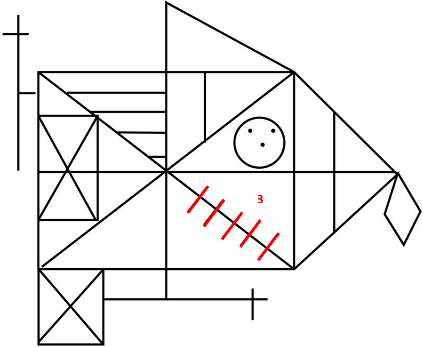 |

Scoring: Rey Complex Figure Task

*Level 7 excellent organization.*

- The rectangle is drawn first
- followed by both center lines,
- all other elements are present and correctly aligned.

*Level 6: conceptual organization*

- Both center lines are drawn as whole single lines, early (i.e., within the first 90 seconds; that is, within the first three colors)
- and completed prior to drawing diagonals and internals sections
- All intersections are correct (i.e., diagonals and centerlines meet at the midpoint junction)
- The majority of the local elements (intern and extern) are present, complete and correctly aligned (i.e., at least 9 out of 12 elements).

*Level 5: Part-configural*

- both centerlines are present
- at least one centerline is drawn as a whole single line,
- the other center line is present and complete
- the proportions of the center lines are connected.
- at least one local element (internal or external) is aligned with vertical centerline, and at least one local element is aligned with horizontal centerline;
- a piecemeal approach is not adopted. That is, at least 75% (i.e. at least 9 local elements are drawn in one colour and not piece by piece in different colors).

*Level 4: piecemeal/ fragmented*

- both centerlines are present
- at least one centerline is drawn as a whole single line, (unless the contour or rectangle is completed first. In this case neither centerline needs to be completed as a whole)
- Most local elements (i.e., at least 9) are present and correctly aligned.
- A piecemeal approach is adopted. That is, less than 9 elements are drawn in one piece (i.e., in one colour).

*Level 3: random organization*

- At least one center line is present (can be fragmented) and at least one internal or external element is present and correctly aligned.

*Level 2: poor organization*

- Any attempt to draw the figure

*Level 1: unrecognizable or substitution*

No attempt is made.

References

Carlson, S. M., & Moses, L. J. (2001). Individual Differences in Inhibitory Control and Children’s Theory of Mind. *Child Development*, *72*(4), 1032–1053. https://doi.org/10.1111/1467-8624.00333

Colantonio, J. A., Bascandziev, I., Theobald, M., Brod, G., & Bonawitz, E. (2024). Predicting Learning: Understanding the Role of Executive Functions in Children’s Belief Revision Using Bayesian Models. *Topics in Cognitive Science*, tops.12749. https://doi.org/10.1111/tops.12749

Davidson, M. C., Amso, D., Anderson, L. C., & Diamond, A. (2006). Development of cognitive control and executive functions from 4 to 13 years: Evidence from manipulations of memory, inhibition, and task switching. *Neuropsychologia*, *44*(11), 2037–2078. https://doi.org/10.1016/j.neuropsychologia.2006.02.006

Diamond, A. (1985). Development of the Ability to Use Recall to Guide Action, as Indicated by Infants’ Performance on AB. *Child Development*, *56*(4), 868. https://doi.org/10.2307/1130099

Gerstadt, C. L., Hong, Y. J., & Diamond, A. (1994). The relationship between cognition and action: Performance of children 312–7 years old on a stroop- like day-night test. *Cognition*, *53*(2), 129–153. https://doi.org/10.1016/0010-0277(94)90068-X

Gopnik, A., & Astington, J. W. (1988). Children’s Understanding of Representational Change and Its Relation to the Understanding of False Belief and the Appearance-Reality Distinction. *Child Development*, *59*(1), 26. https://doi.org/10.2307/1130386

Kerns, K. A., & McInerney, R. (2007). *Preschool Tasks* [Software]. Victoria, BC, Canada: University of Victoria.

Kimura, K. (2020). *Belief Revision in Children and Adults* [University of California]. https://escholarship.org/content/qt5x49v8dk/qt5x49v8dk_noSplash_7e5bb8fdd6047f3db8b1dfe9abf207b8.pdf

Petermann. (2009). *Wechsler preschool and primary scale of intelligence – III (WPPSI-III; German Version).* Pearson Assessment.

Stöttinger, E., Guay, C. L., Danckert, J., & Anderson, B. (2018). Updating impairments and the failure to explore new hypotheses following right brain damage. *Experimental Brain Research*, *236*(6), 1749–1765. https://doi.org/10.1007/s00221-018-5259-6

Stöttinger, E., Sepahvand, N. M., Danckert, J., & Anderson, B. (2016). Assessing perceptual change with an ambiguous figures task: Normative data for 40 standard picture sets. *Behavior Research Methods*, *48*(1), 201–222. https://doi.org/10.3758/s13428-015-0564-5
